# Comprehensive analysis of a pyroptosis-related gene signature of clinical and biological values in spinal cord injury

**DOI:** 10.3389/fneur.2023.1141939

**Published:** 2023-05-19

**Authors:** Pingping Zhang, Jianping Zhang, Wenjuan Kou, Guangjin Gu, Yaning Zhang, Weihan Shi, Pengcheng Chu, Dachuan Liang, Guangwei Sun, Jun Shang

**Affiliations:** ^1^Department of Orthopedics, Seventh Affiliated Hospital of Shanxi Medical University, Linfen People's Hospital, Linfen, Shanxi, China; ^2^Department of Orthopedics, Tianjin Medical University General Hospital, Tianjin, China; ^3^School of Pharmaceutical Sciences and Research Center of Basic Medical Sciences, Tianjin Medical University, Tianjin, China; ^4^Department of Disinfection Monitoring, Yongji Disease Control and Prevention Center, Yongji, Shanxi, China; ^5^Department of Scientific Research Management, Shanxi Medical College Seventh Affiliated Hospital, Linfen People's Hospital, Linfen, Shanxi, China

**Keywords:** spinal cord injury, peripheral blood, biomarkers, bioinformatics analysis, clinical examination, immune infiltration

## Abstract

**Background:**

Since some of the clinical examinations are not suitable for patients with severe spinal cord injury (SCI), blood biomarkers have been reported to reflect the severity of SCI. The objective of this study was to screen out the potential biomarkers associated with the diagnosis of SCI by bioinformatics analysis.

**Methods:**

The microarray expression profiles of SCI were obtained from the Gene Expression Omnibus (GEO) database. Core genes correlated to pyroptosis were obtained by crossing the differential genes, and module genes were obtained by WGCNA analysis and lasso regression. The immune infiltration analysis and GSEA analysis revealed the essential effect of immune cells in the progression of SCI. In addition, the accuracy of the biomarkers in diagnosing SCI was subsequently evaluated and verified using the receiver operating characteristic curve (ROC) and qRT-PCR.

**Results:**

A total of 423 DEGs were identified, among which 319 genes were upregulated and 104 genes were downregulated. Based on the WGCNA analysis, six potential biomarkers were screened out, including LIN7A, FCGR1A, FGD4, GPR27, BLOC1S1, and GALNT4. The results of ROC curves demonstrated the accurate value of biomarkers related to SCI. The immune infiltration analysis and GSEA analysis revealed the essential effect of immune cells in the progression of SCI, including macrophages, natural killer cells, and neutrophils. The qRT-PCR results verified that FGD4, FCAR1A, LIN7A, BLOC1S1, and GPR27 were significantly upregulated in SCI patients.

**Conclusion:**

In this study, we identified and verified five immune pyroptosis-related hub genes by WGCNA and biological experiments. It is expected that the five identified potential biomarkers in peripheral white blood cells may provide a novel strategy for early diagnosis.

## 1. Introduction

Spinal cord injury (SCI) is a permanent traumatic disease of the central nervous system (CNS), with high mortality, disability, and complications (1, 2). A variety of clinical examinations can contribute to SCI diagnosis. However, some of the clinical examinations such as magnetic resonance imaging (MRI) are not suitable for some patients with special conditions like metal puncture or metal implant. Researchers began to study blood biomarkers to predict the severity of SCI, based on which we try to find out a new diagnostic strategy for human SCI by screening out the potential biomarkers in peripheral blood (3, 4).

Since 1981, several studies have found that some biomarkers were related to SCI severity which could be used to predict outcomes and confirm the diagnosis (5–8). Most of them used proteomics to identify serum and cerebrospinal fluid (CSF) biomarkers. The early diagnosis and evaluation usually predict the prognosis. However, it is impractical to collect CSF samples from acute SCI patients whose vital signs are unstable. Because of the destruction of the blood–spinal cord barrier after SCI, some biomarkers can also be detected in peripheral blood. Thus, peripheral blood samples can be used as an alternative to CSF samples. It should be noted that not all the biomarkers in CSF are applicable in peripheral blood samples. In addition, circulating immune cells are major concerns that can reflect the immune response related to SCI (3, 9).

With the development of bioinformatics analysis technology, many biomarkers in blood samples have been screened out to guide diagnosis and prognosis clinically. Overall, in this study, we used RNA-sequencing data (GSE151371 from the GEO database, https://www.ncbi.nlm.nih.gov/geo/query/acc.cgi?acc=GSE151371) to analyze global gene expression in peripheral white blood cells in order to identify the potential biomarkers associated with the early diagnosis for human SCI in peripheral blood samples.

## 2. Methods

### 2.1. Data acquisition

The microarray expression profiles of SCI were obtained from the GEO (Gene Expression Omnibus, http://www.ncbi.nlm.nih.gov/geo/) database with the serial number GSE151371. In total, 58 samples (38 from SCI patients, 10 from healthy controls, and 10 from trauma controls with non-CNS injuries) were included in the GSE151371 dataset.

### 2.2. Identification and functional enrichment analysis of DEGs

Differential gene expression between the two teams of samples in GSE151371 was analyzed by applying the limma package of R software. “Adjusted |log2 (FC)| > 1.5 and *P* < 0:05” (10, 11) was described as the screening conditions for differential borderline gene expression. PCA graphs were drawn using the ggord package of R software, and the heat map is displayed through the pheatmap package of R software. To uncover the functions of the DEGs and distinguish the crucial pathways related to the DEGs, the Gene Ontology (GO) analyses, Kyoto Encyclopedia of Genes and Genomes (KEGG) enrichment analysis, and gene set enrichment analysis (GESA) were operated with the “clusterProfiler” package in R. A *p*-value of < 0.05 was considered to be statistically significant (12). The C7 immunologic signatures gene set database was freely available from the Molecular Signature Database (MsigDB) as a reference for the KEGG analysis. The gene set arrangement was performed 1,000 times per analysis. To identify enriched function terms, GSEA with a false discovery rate (FDR) of < 0.25 and a threshold of a *p*-value of < 0.05 was executed utilizing the Molecular Signatures Database (MSigDB).

### 2.3. Venn diagrams of DEGs and pyroptosis-related genes

This article incorporated 81 genes associated with pyroptosis in accordance with previous studies (13–15). The Venn diagram drawing tools (http://bioinformatics.psb.ugent.be/webtools/Venn/) were employed to create Venn diagrams between DEGs and ferroptosis-related genes.

### 2.4. WGCNA analysis

A co-expression network of the chosen dataset was constructed through the “WGCNA” package in the R program. First, the outliers were screened out to obtain a more constant model, and we chose the appropriate soft threshold β. Thereafter, the topological overlap matrix (TOM) was additionally formed, and hierarchical clustering was used to generate a gene-level clustering tree. We chose module membership (MM) and gene significance (GS) to verify the relationship between genes and clinical information in order to affirm the key modules and genes.

### 2.5. Acquisition of biomarkers in SCI

Core genes correlated to pyroptosis were obtained by crossing the differential genes and module genes obtained by the WGCNA analysis. Thereafter, the Least Absolute Shrinkage and Selection Operator (LASSO) algorithm of the glmnet package in the R program is employed to select the cordial genes. In order to choose the candidate genes, we utilized the LASSO algorithm in the R program package glmnet. The diagnostic value of the obtained genes was visualized by the plotted ROC curve. The genes obtained eventually are of significant value in the diagnosis of SCI.

### 2.6. Assessment of immune cell infiltration and association analysis between hub genes and infiltrating immune cells

The relative infiltration levels of 16 immune cells in the GSE151371 dataset were measured with the ssGSEA algorithm (16). Violin plots were plotted to show the differential expression levels of 16 immune infiltrating cells. Moreover, the Spearman correlation between hub genes and 16 immune infiltrating cells was calculated, and the results were visualized using the ggplot2 package in the R program (v 3.1.1).

### 2.7. qRT-PCR

Peripheral blood samples were taken from healthy people and patients with spinal cord injuries. Total RNA was extracted from peripheral blood to synthesize cDNA according to the manufacturer's instructions (Takara Biotech Co., Beijing, China). qRT-PCR was run on a LightCycler 96 (Roche Life Sciences, Swiss, Basel) using a real-time PCR mix (Cowin Biotech, Taizhou, Jiangsu, China). The 2–ΔΔCt method was applied to evaluate gene expression relative to GAPDH and DEGs. The independent experiment was repeated three times (the Primer sequence fragments of RNAs are displayed in Table 1).

**Table 1:** Sequence fragments of RNA.

	**Forward**	**Reverse**
LIN7A	5′-GCAACAGCAAAGGCAACAGT-3′	5′-CTCTTTTGAGGCCTCCGTGT-3′
FCGR1A	5′-GCCACAGAGGATGGAAATGT-3′	5′-CATGAAACCAGACAGGAGTGG-3′
FGD4	5′-TCAGATCTCATCAGTCGCTTTG-3′	5′-ACAGCAGACTCTTTCTTCAAATCA-3′
GPR27	5′-GCCTCCGTGTGGCTGACCTTC-3′	5′- ACCAATGCCTTTCAGGTCGCAG-3′
BLOC1S1	5′-CCCAATTTGCCAAGCAGACA-3′	5′-CATCCCCAATTTCCTTGAGTGC-3′
GALNT4	5′-GGCCTATATCTTCGTGGAGCTC-3′	5′-CCTGCGGAGGCATGAAAA-3
GAPDH	5′-CTGGGCTACACTGAGCACC-3′	5′-AAGTGGTCGTTGAGGGCAATG-3′

### 2.8. Statistical analysis

All statistical analyses and data calculations were conducted with R version 3.4.3 (http://www.r-projec-t.org). Between different groups, comparisons were conducted by applying the independent Student's *t*-test. Two-tailed *p*-values of < 0.05 were identified as statistically significant.

## 3. Results

### 3.1. Identification of DEGs

**Figure 1:**
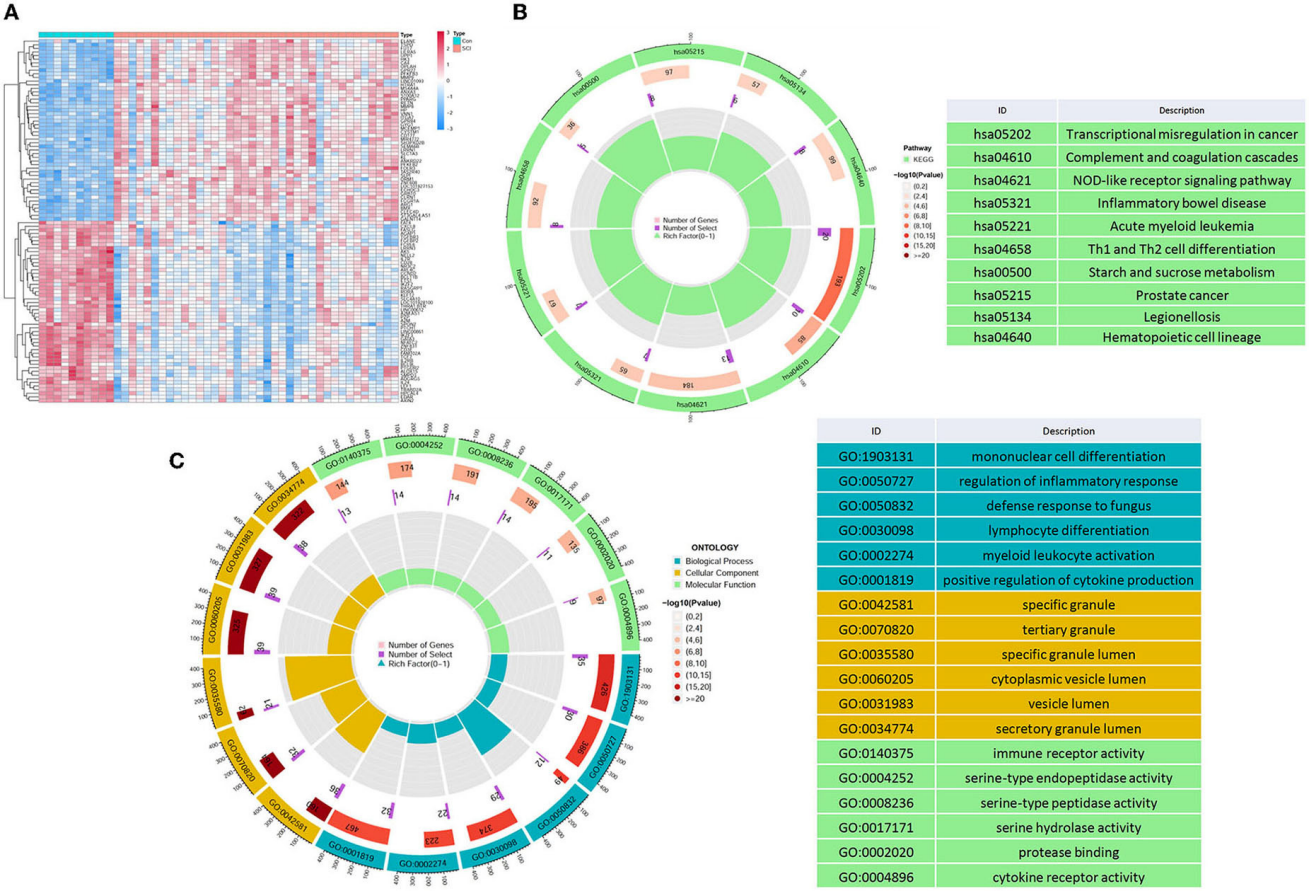
Differential mapping and functional enrichment analysis of SCI. **(A)** Thermal plots of DEGs. Through the differential analysis of microarray data, a sum of 423 DEGs were identified, including 319 upregulated genes and 104 downregulated genes, **(B)** KEGG, and **(C)** GO enrichment analysis of DEGs in SCI.

Through differential analysis, we detected a total of 423 DEGs in GSE151371, which are displayed in the volcano plot (Figure 1A), including 319 upregulated genes and 104 downregulated genes. To investigate the biological functions of DEGs, GO (Figure 1B) and KEGG term enrichment (Figure 1C) analyses were employed to gain insights into all the upregulated and downregulated DEGs, respectively.

### 3.2. Acquisition of pyroptosis-related DEGs

**Figure 2:**
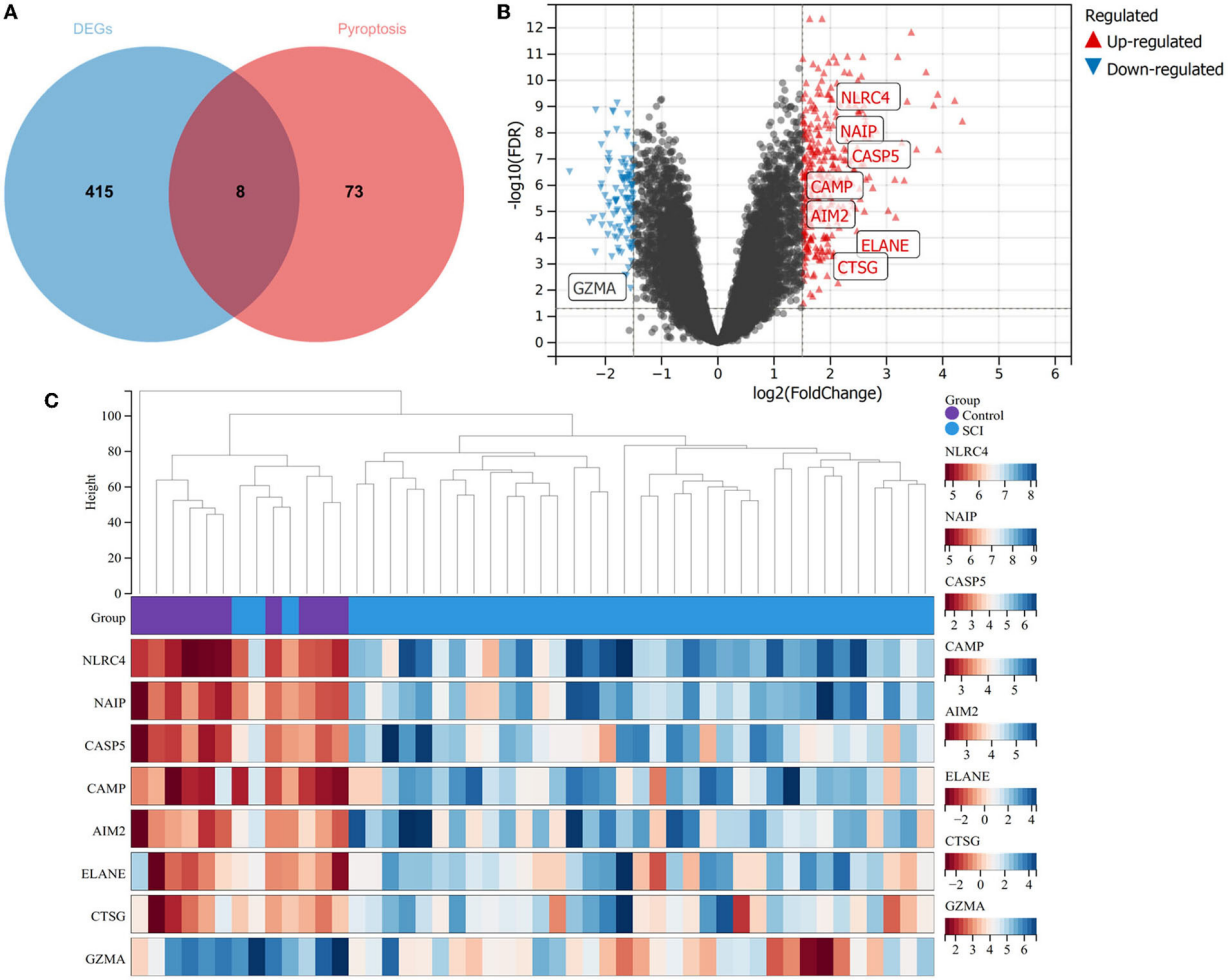
Identification of pyroptosis-related DEGs. **(A)** Venn diagram between DEGs and pyroptosis. **(B)** Volcano plot for pyroptosis-related DEGs between SCI patients and healthy controls. **(C)** Thermal plots of pyroptosis DEGs between healthy controls and SCI patients.

It has been proved that pyroptosis is a significant factor in SCI. Thus, we found 81 pyroptosis-related genes and operated intersections with DEGs (Figure 2A). The seven upregulated genes, including, NLRC4, NAIP, CASP5, CAMP, AIM2, ELANE, and CTSG, and one downregulated gene, namely, GZMA (Figures 2B, C), were obtained.

### 3.3. Acquisition of pyroptosis-related gene modules

**Figure 3:**
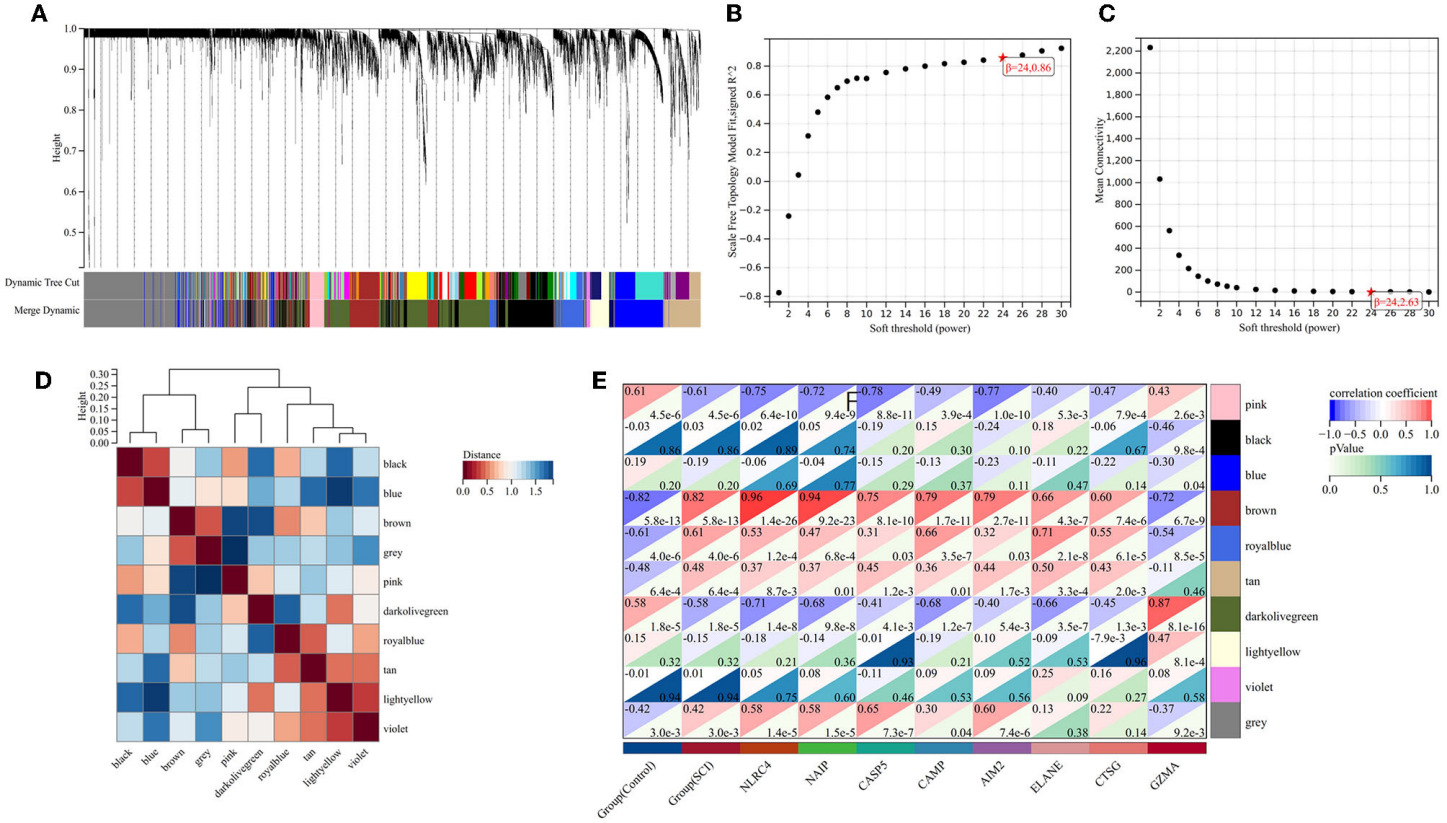
Weighted gene co-expression network analysis. **(A)** Genetic tree diagram. **(B, C)** The analysis plot of network topologies under different soft threshold powers showed that the correlation coefficient was 0.86, and the optimal soft thresholding power was 24. **(D)** Module feature vector clustering heat map. **(E)** Heat map about relationships between differential pyroptosis-related genes and module signature genes. The brown module was notably related to pyroptosis.

**Figure 4:**
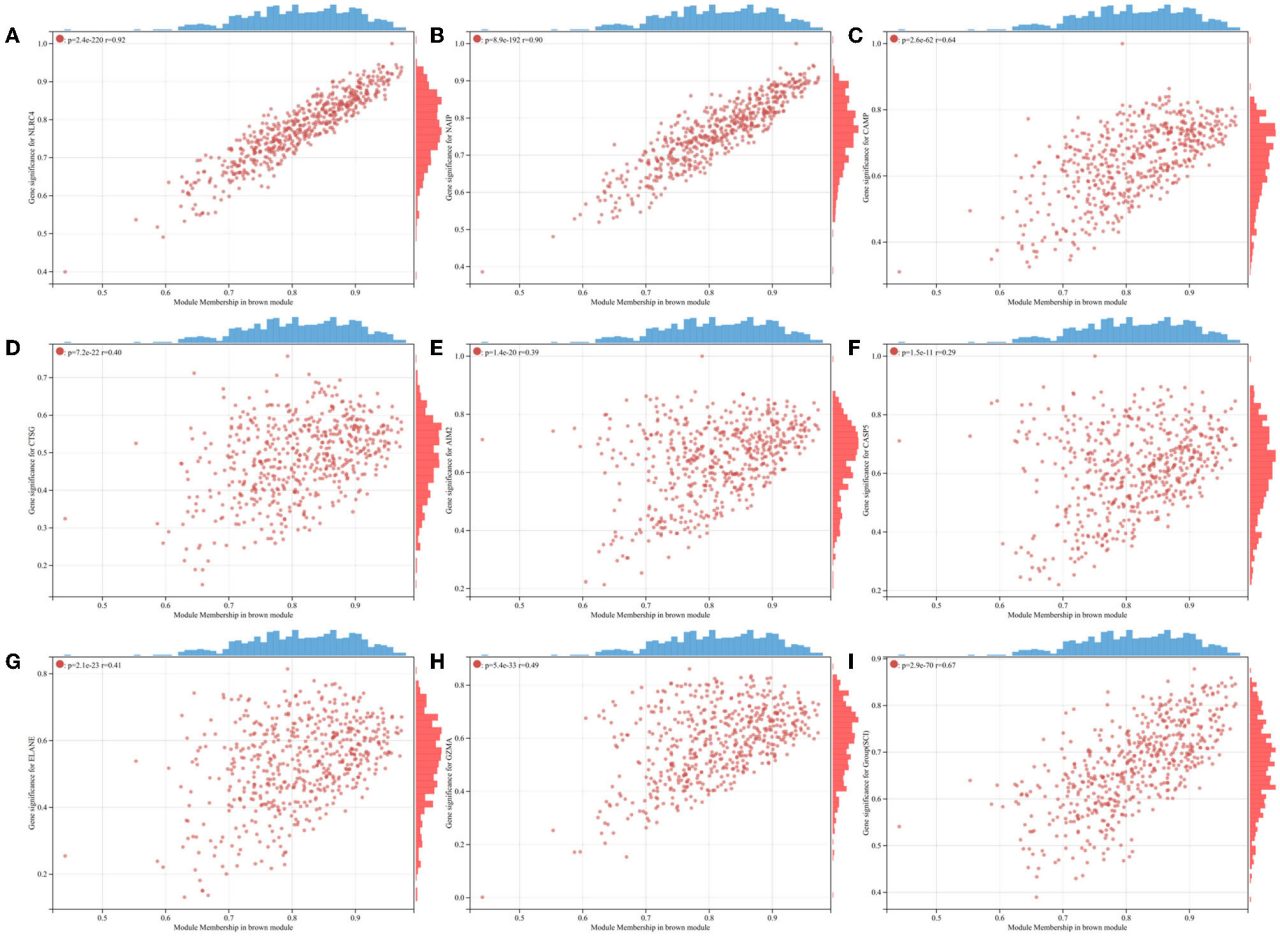
Scatterplots of GS for individual pyroptosis genes vs. MM in the brown module. **(A)** NLRC4. **(B)** NAIP. **(C)** CAMP. **(D)** CTSG. **(E)** AIM2. **(F)** CASP5. **(G)** ELANE. **(H)** GZMA. **(I)** Group(SCI).

To probe the genes associated with the pyroptosis module, we performed the WGCNA analysis on the GSE151371 dataset. By means of the R program package WGCNA, we created the model (Figure 3A). Through the usage of the pick soft threshold function, we screened the optimal soft threshold for this model to be 24; meanwhile, R^2^ was 0.86 (Figure 3B) and average connectivity was 2.36 (Figure 3C). By blending analogous modules, this model created ten separate modules (Figure 3D). Then, we associated the co-expression module and identified that the brown module met our needs better (Figure 3E) shows a heat map of the association between pyroptosis and the module, and Figure 4 shows a scatter plot of the association between MM and GS).

### 3.4. Acquirement of cordial biomarkers for SCI

**Figure 5:**
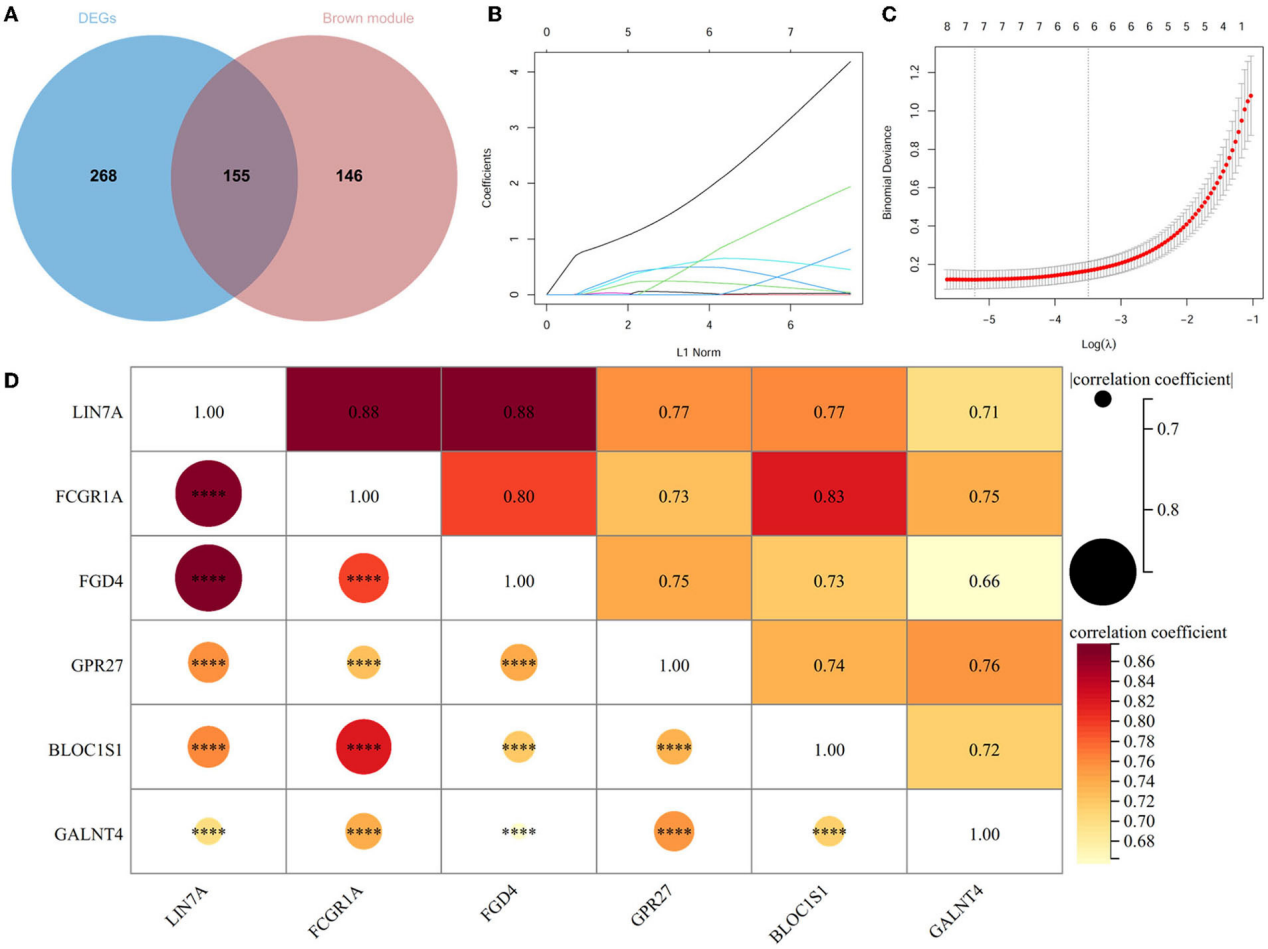
Acquirement of candidate genes. **(A)** Venn diagram between DEGs and brown modules of WGCNA. **(B)** Plot of LASSO coefficient for cordial genes. **(C)** Validation of LASSO regression analysis. **(D)** Heat map for relevance analysis of six hub genes. ^****^P < 0.0001.

**Figure 6:**
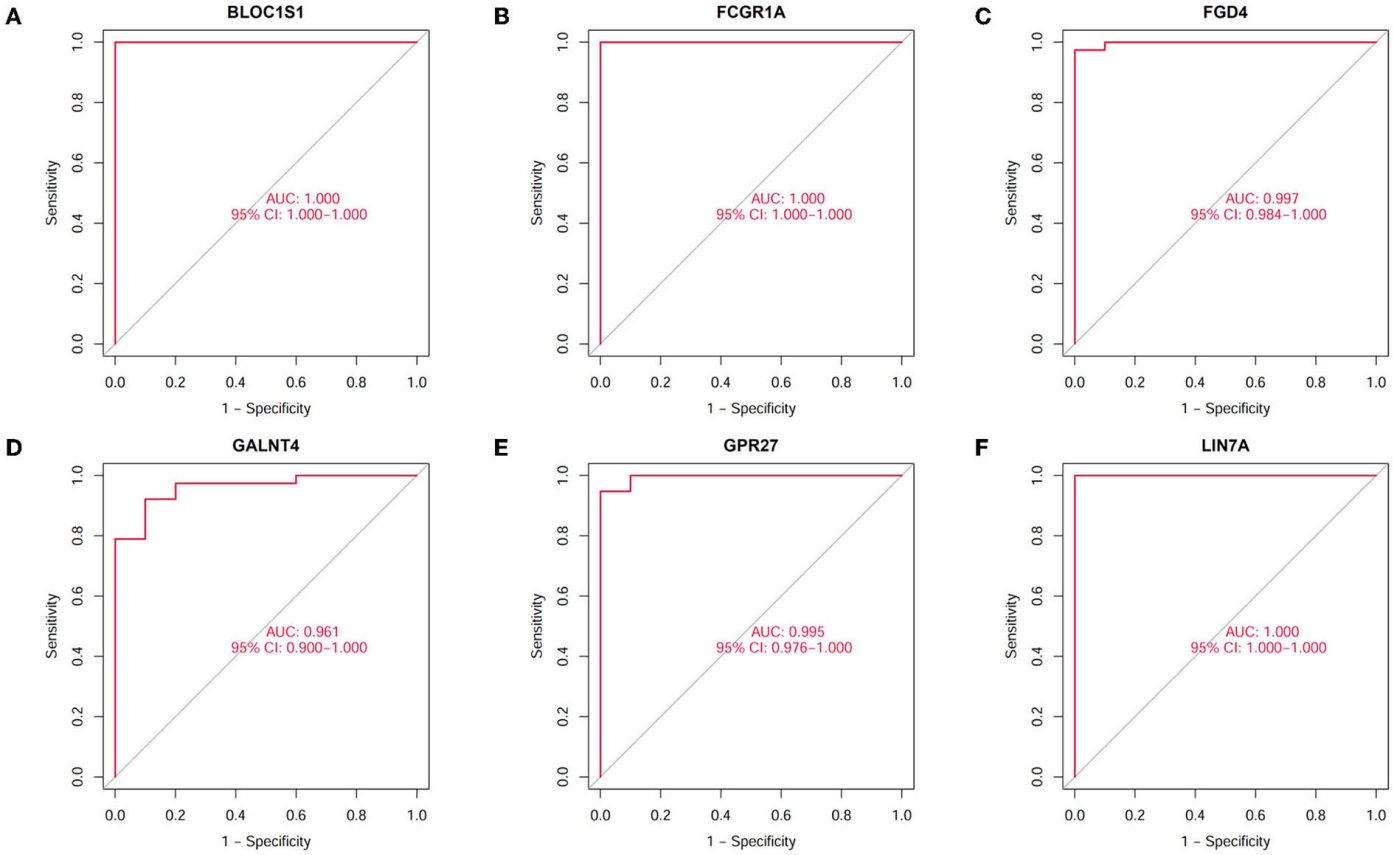
Diagnostic value of hub genes in SCI. The ability to distinguish spinal cord injury from healthy controls was assessed by the ROC curve and AUC statistics. **(A)** Validation of BLOC1S1. **(B)** Validation of FCGR1A. **(C)** Validation of FGD4. **(D)** Validation of GALNT4. **(E)** Validation of GPR27. **(F)** Validation of LIN7A.

**Figure 7:**
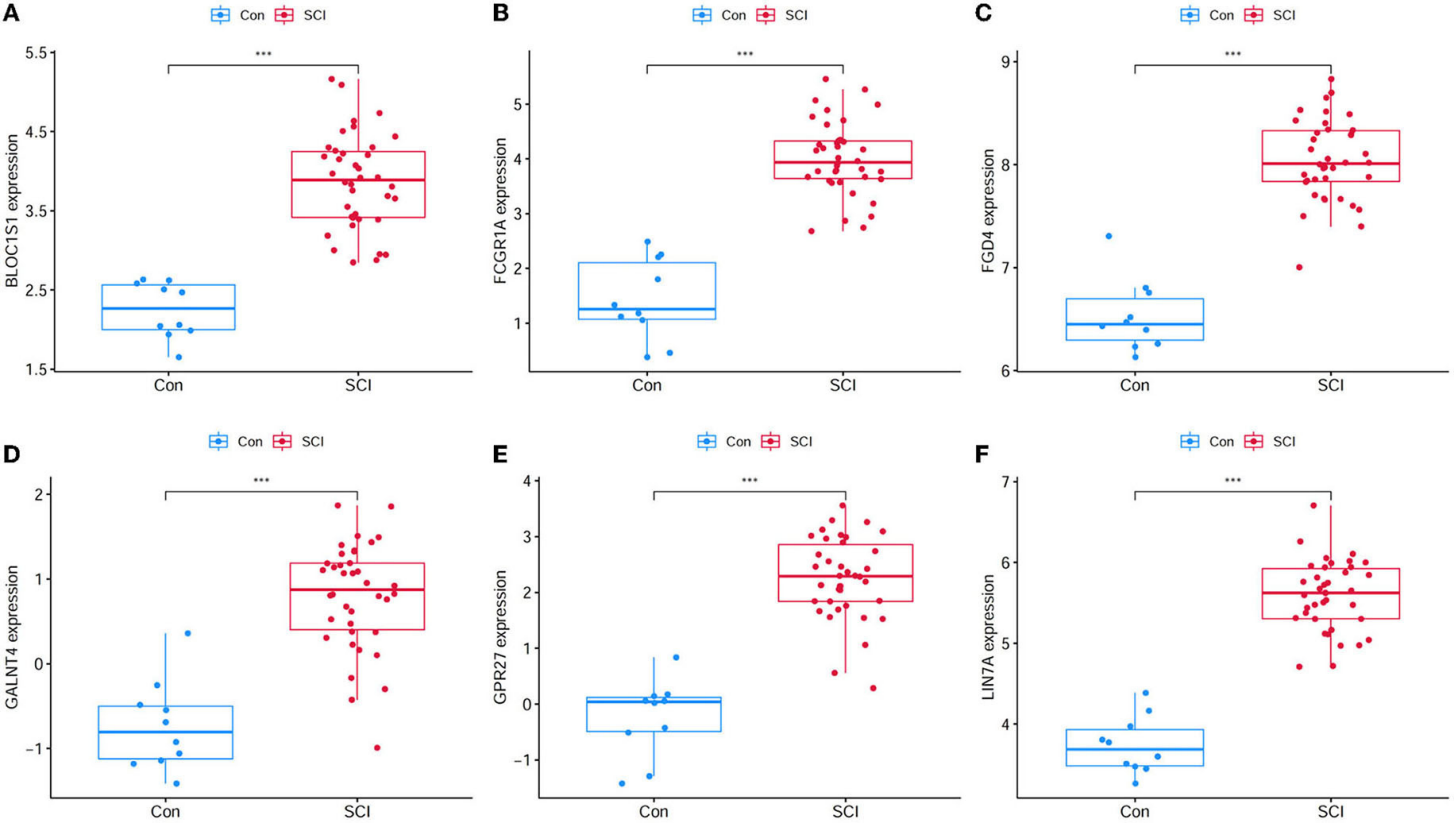
Gene expression level of candidate genes between healthy controls and SCI samples. **(A)** BLOC1S1 was significantly higher expression in SCI compared with healthy control. **(B)** FCGR1A was significantly higher expression in SCI compared with healthy control. **(C)** FGD4 was significantly higher expression in SCI compared with healthy control. **(D)** GALNT4 was significantly higher expression in SCI compared with healthy control. **(E)** GPR27 was significantly higher expression in SCI compared with healthy control. **(F)** LIN7A was significantly higher expression in SCI compared with healthy control. ^***^P < 0.001.

A total of 423 DEGs were identified in GSE151371 using the limma package. In total, 155 related genes were acquired by crossing with the gene module obtained using WGCNA (Figure 5A). Thereafter, 155 genes were imported into LASSO using a machine-learning method to obtain gene markers with a high diagnostic value. We acquired six candidate markers through LASSO coefficient profiles (Figure 5B) and validation (Figure 5C), including, “LIN7A,” “FCGR1A,” “FGD4,” “GPR27,” “BLOC1S1,” and “GALNT4.” The six obtained genes were subjected to correlation analysis (Figure 5D), and all six genes were highly correlated with each other. The effectiveness of hub genes was verified by the ROC curve (Figure 6), and their diagnostic sensitivity for SCI was evaluated by the values of the area under the curve for the six hub genes. In all six candidate genes, the AUC values > 0.95 demonstrated the crucial diagnostic value of these genes for SCI. We made use of box plots to certify the expression levels of the six candidate genes. Figures 7A–F exhibits notably higher expression levels of LIN7A (*P* = 4.43E-13), FCGR1A (*P* = 1.24E-11), FGD4 (*P* = 1.44E-11), GPR27 (*P* = 1.0E-10), BLOC1S1 (*P* = 6.58E-9), and GALNT4 (*P* = 1.18E-7) in SCI tissues than in healthy controls.

### 3.5. Correlation between hub genes and immune cell infiltration

**Figure 8:**
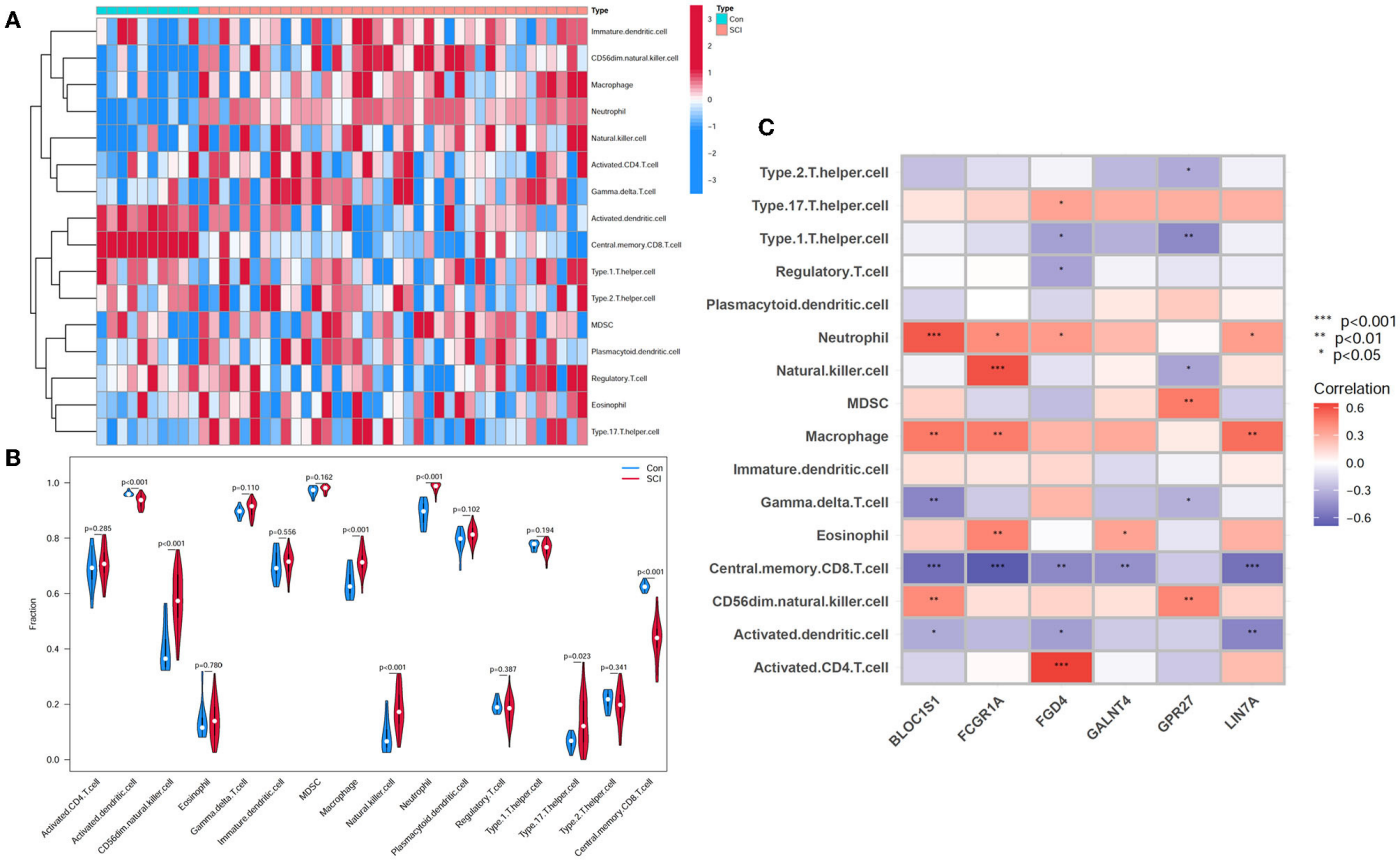
Analysis of immune landscape correlated with SCI. **(A)** Heatmap. **(B)** Violin plot demonstrating the distribution of 16 types of immune cells in SCI samples and healthy control. **(C)** Correlation between immune cell infiltration and six hub genes.

To investigate the relationship between SCI and healthy controls in terms of immune cell infiltration, the ssGSEA algorithm was applied. Figure 8A shows the distribution of 16 immune cells in the GSE151371 sample. Analysis of immune cell infiltration revealed a remarkably higher infiltration of CD56dim natural killer cells, macrophages, neutrophils, natural killer cells, and type 17 T helper cells in SCI than in the healthy tissue, and a notably lower infiltration of central memory CD8 T cell and activated dendritic cell, implying that these cells are vital in the development of SCI (Figure 8B). A correlation analysis of 16 immune cells with hub genes revealed that CD56dim natural killer cells were positively correlated with BLOC1S1 (cor = 0.419; *P* < 0.01) and GPR27 (cor = 0.44; *P* < 0.01). Macrophages were positively related with LIN7A (cor = 0.518; *P* = 0.001), FCGR1A (cor = 0.469; *P* = 0.003), and BLOC1S1 (cor = 0.479; *P* = 0.003). Natural killer cells had a positive correlation with FCGR1A (cor = 0.603; *P* < 0.001) and a negative correlation with GPR27 (cor = −0.370; *P* < 0.05). Neutrophil was positively associated with BLOC1S1 (cor = 0.583; *P* < 0.001), LIN7A, FCGR1A, and FGD4 (all *P* < 0.05). Type 17 T helper cells had a positive relationship with FGD4 (cor = 0.334; *P* < 0.05). Activated dendritic cell had a negative relationship with LIN7A (cor = −0.506; *P* < 0.01), FGD4, and BLOC1S1(all *P* < 0.05). Central memory CD8 T cell had a negative correlation with LIN7A (cor = −0.506; *P* < 0.001), FCGR1A (cor = −0.690; *P* < 0.001), BLOC1S1(cor = −0.601; *P* < 0.001), FGD4 (cor = −0.491; *P* < 0.01), and GALNT4 (cor = −0.453; *P* < 0.01) (Figure 8C). These results further indicated that these immune cells were involved in the development of spinal cord injury and played a significant role in it.

### 3.6. Enrichment analysis of immune signature gene sets

**Figure 9:**
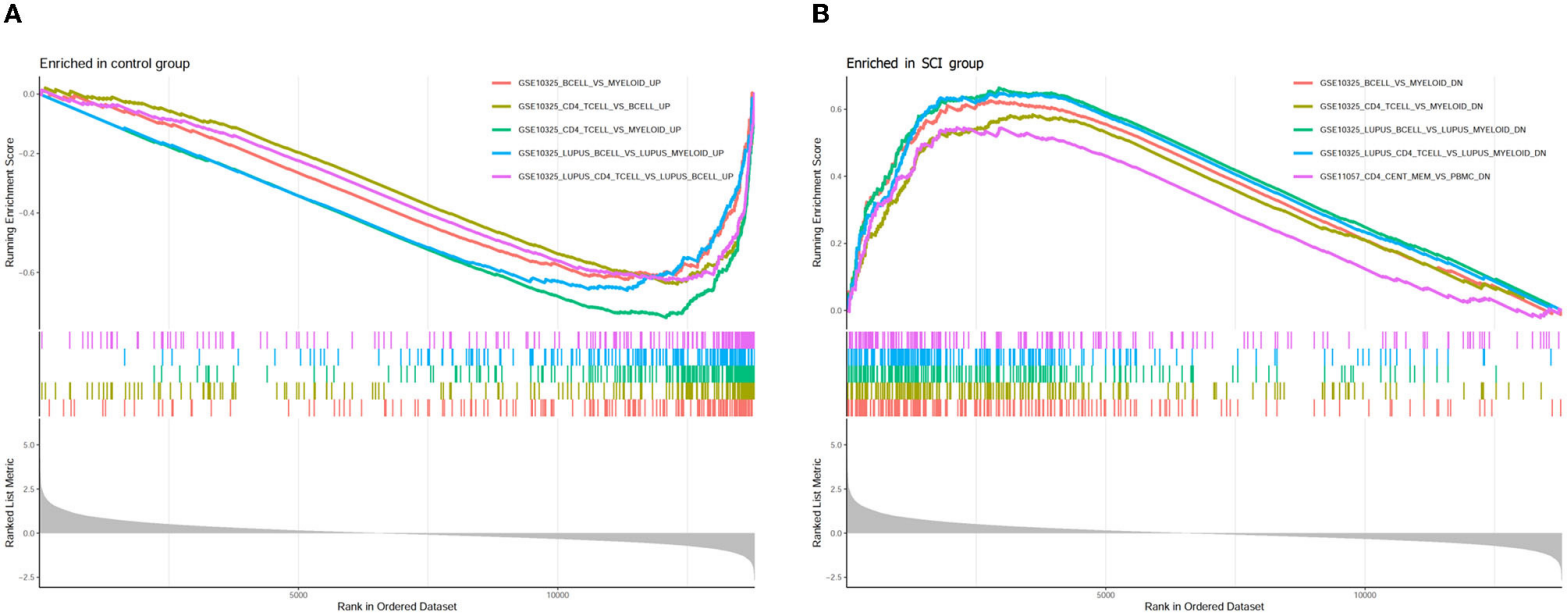
Enrichment plot for GSEA immunologic signature database in **(A)** healthy control and **(B)** SCI samples.

In order to explore the underlying immune mechanism in the development of spinal cord injury, the immune marker gene set in the MsigDB database was applied as the reference for the GSEA of DEGs. In total, 1,238 gene sets were remarkably harvested (|normalized enriched score (NES)| > 1; FDR q-value < 0.05) (|NES| > 1; FDR q-value < 0.05). These gene sets were predominantly enriched in the B cells, CD4^+^ T cells, lupus erythematosus B cells, and lupus erythematosus CD4 T cells in the control group. In addition, these gene sets were mostly enriched dominant in myeloid cells, systemic lupus erythematosus myeloid cells, and peripheral blood mononuclear cells (PBMCs) in the SCI group. The top 10 enriched gene sets are recorded in Table 2. It can be concluded that the immune-related genes perform a vital part in the initiation and development of SCI (Figure 9).

**Table 2:** Top 10 significant immunologic signatures enriched by DEGs in GSEA.

**Gene set name**	**NES**	**p-value**	**NOM p-val**	**FDR q-val**
GSE10325 B cell vs. myeloid down	2.666302797	1.00E-10	3.72E-09	2.55E-09
GSE10325 B cell vs. myeloid up	−2.987569696	1.00E-10	3.72E-09	2.55E-09
GSE10325 CD4 T cell vs. B cell up	−3.076363212	1.00E-10	3.72E-09	2.55E-09
GSE10325 CD4 T cell vs. myeloid down	2.464848701	1.00E-10	3.72E-09	2.55E-09
GSE10325 CD4 T cell vs. myeloid up	−3.627653133	1.00E-10	3.72E-09	2.55E-09
GSE10325 Lupus B cell vs. lupus myeloid down	2.83252892	1.00E-10	3.72E-09	2.55E-09
GSE10325 Lupus B cell vs. lupus myeloid up	−3.155880553	1.00E-10	3.72E-09	2.55E-09
GSE10325 Lupus CD4 T cell vs. lupus B cell up	−3.008565951	1.00E-10	3.72E-09	2.55E-09
GSE10325 Lupus CD4 T cell vs. lupus myeloid down	2.764655436	1.00E-10	3.72E-09	2.55E-09
GSE11057 CD4 T cell memory vs. PBMCs down	2.301034264	1.00E-10	3.72E-09	2.55E-09

DEGs, differentially expressed genes; GSEA, gene set enrichment analysis; NES, normalized enrichment score; NOM, nominal; FDR, false discovery rate.

### 3.7. Expression level of cordial DEGs

**Figure 10:**
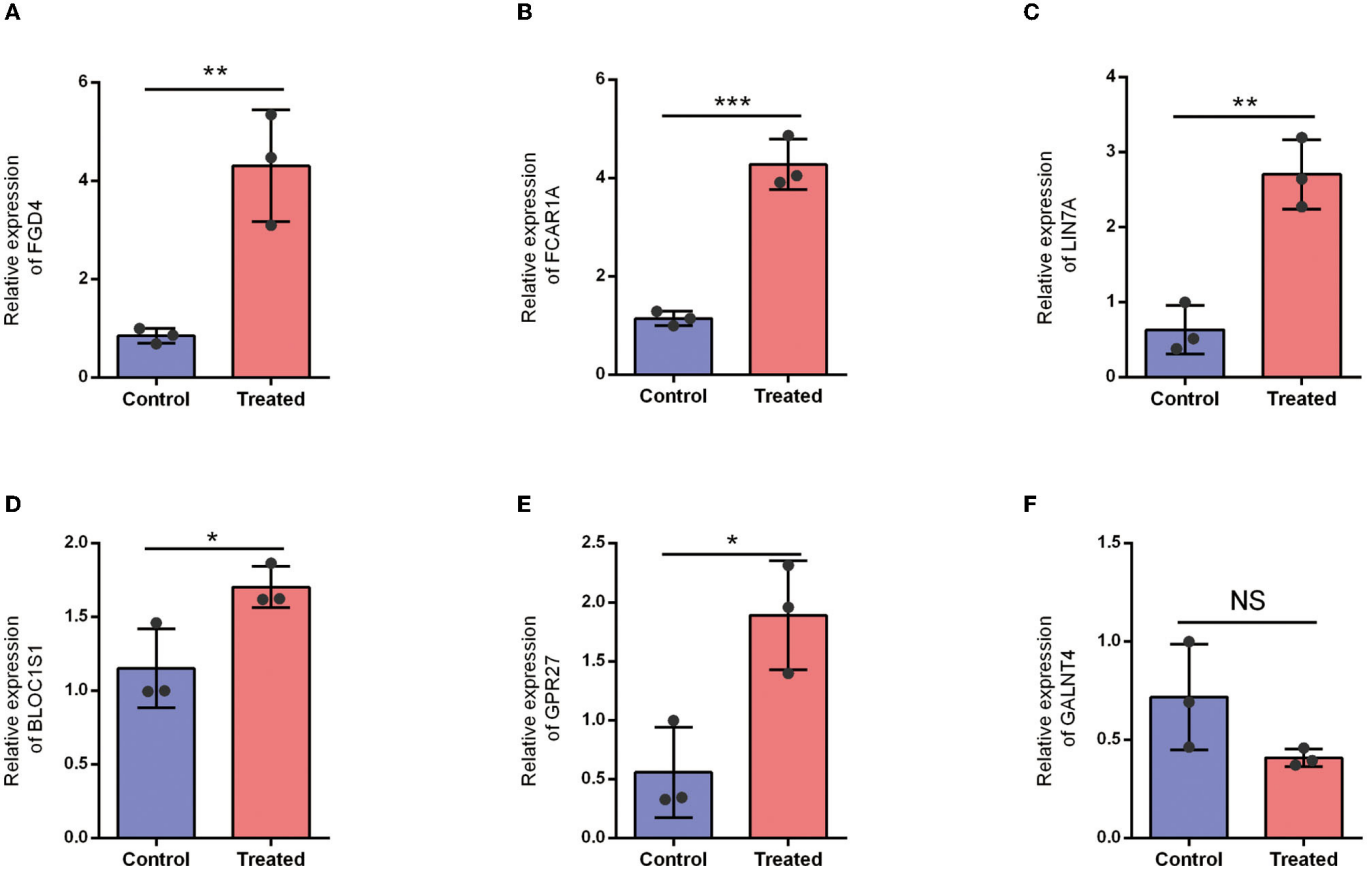
Expression level of hub DEGs in healthy control and SCI patients. **(A)** Expression level of FGD4. **(B)** Expression level of FCAR1A. **(C)** Expression level of LIN7A. **(D)** Expression level of BLOC1S1. **(E)** Expression level of GPR27. **(F)** Expression level of GALNT4. NS: *P* > 0.05; ^*^P < 0.05; ^**^P < 0.01; ^***^P < 0.001.

To further validate the correctness of the analyzed outcomes, the expression of six DEGs was identified using qRT-PCR. The outcomes displayed that five DEGs remarkably associated with SCI were upregulated in SCI patients. Furthermore, the results demonstrated that FGD4, FCAR1A, LIN7A, BLOC1S1, and GPR27 were significantly upregulated in an SCI patient (Figure 10).

## 4. Discussion

According to the results of this study, by a series of bioinformatics analysis methods, we screened out six potential biomarkers correlated with SCI, including “LIN7A,” “FCGR1A,” “FGD4,” “GPR27,” “BLOC1S1,” and “GALNT4.” LIN7A is wellstudied in malignant tumors, such as breast carcinomas, ovarian cancer, and hepatocellular carcinoma (17–19), whose encoded protein is a small scaffold protein related to PDZ domain binding and L27 domain binding. LIN7A plays a significant role in installing and retaining the unbalanced distribution of receptors and channels at the polarized cells' plasma membrane, among whose related pathways are the neurotransmitter release cycle and protein–protein interactions at synapses. Although there is no relevant research on SCI, LIN7A has been reported to regulate cerebral cortex development (20), which may have the capacity to link synaptic vesicle exocytosis with cell adhesion in the brain. Furthermore, more and more evidence has revealed the potential relationship between LIN7 and neuronal disorders including autism, Huntington's disease, and attention-deficit/hyperactivity disorder (ADHD) (21–23). It is reasonable to believe that LIN7A can reflect the severity of SCI.

FCGR1A-encoded protein is a high-affinity Fc gamma receptor (FcγR) that plays a significant role in immune feedback. FcγR was also identified to distribute on neurons of the central and peripheral nervous system which play roles in various neurological diseases, such as stroke, Parkinson's disease, and Alzheimer's diseases (24–26). A study demonstrated that FCGR1A transcripts in peripheral blood could be used as a predictive marker of intrathoracic tuberculosis (27). Although there are no relevant reports, FCGR1A could also reflect the immune response after SCI.

FGD4 plays a crucial role in regulating the actin cytoskeleton and cell shape. In the study of Charcot–Marie-Tooth Type 4H disease, one of the most common inherited neurological disorders, FGD4 is proved crucial for accurate myelin maintenance and correct nerve development (28). Frabin is the gene product of FGD4, and it has been reported that the loss of Frabin/FGD4 will cause demyelination of peripheral nerves (29). At present, there is no research on FGD4 on CNS; however, it might reflect the pathological changes of demyelination after SCI.

Spinal cord injury is a pathological incident that triggers several neuropathological conditions, leading to the initiation of neuronal damage with several pro-inflammatory mediators' release. However, pyroptosis is recognized as a new programmed cell death mechanism regulated by the stimulation of caspase-1 and/or caspase-11/-4/-5 signaling pathways with a series of inflammatory responses. Multiple pieces of evidence have illustrated that pyroptosis plays significant roles in cell swelling, plasma membrane lysis, chromatin fragmentation, and intracellular pro-inflammatory factors including IL-18 and IL-1β release, after spinal cord injury (30). The obtained biomarkers related to pyroptosis after spinal cord injury can further target the biological process, thereby inhibiting pyroptosis and providing new strategies for the treatment of spinal cord injury.

Although there were no statistical differences in the expression of GPR27 and BLOC1S1, they still showed a trend of upregulation after SCI. GPR27 is one of the members of G protein-coupled receptors (GPCRs), which is highly conserved in vertebrate evolution and mainly expressed in the brain (31). There is evidence reporting that GPR27 plays an important role in nervous system diseases such as schizophrenia and autism (31). BLOOC1S1 regulates the biosynthesis of the endosomal–lysosomal system, which is essential for protein transport within cells (32). A recent study found that BLOC1S1 mutation is associated with leukodystrophy, showing neural phenotypes including abnormal myelination, intellectual disability, leukodystrophy, optic atrophy, and severe global developmental delay (33). Therefore, GPR27 and BLOC1S1 also show a close relationship to CNS development and diseases, which can reflect the diagnosis and prognosis for SCI to a certain extent.

## 5. Conclusion

Taken together, in this study, we identified and verified five immune pyroptosis-related hub genes by WGCNA and biological experiments. It is expected that the five identified potential biomarkers in peripheral white blood cells may provide a novel strategy for early diagnosis through peripheral blood samples.

## Data availability statement

The original contributions presented in the study are included in the article/Supplementary material, further inquiries can be directed to the corresponding authors.

## Ethics statement

The studies involving human participants were reviewed and approved by Seventh Affiliated Hospital of Shanxi Medical University; Linfen People's Hospital. The patients/participants provided their written informed consent to participate in this study. Written informed consent was obtained from the individual(s) for the publication of any potentially identifiable images or data included in this article.

## Author contributions

PZ and JS developed the experimental design. PZ, JZ, and WK directed the overall research. JZ, WK, and GG performed the experiments. YZ, PC, DL, and GS analyzed the data and prepared the figures. All authors contributed to the article and approved the submitted version.

## Supplementary material

Supplementary Table 1 One thousand two hundred thirty-eight immune gene sets in the MSigDB.
